# HPV type distribution in invasive cervical cancers in Italy: pooled analysis of three large studies

**DOI:** 10.1186/1750-9378-7-26

**Published:** 2012-10-12

**Authors:** Paolo Giorgi Rossi, Mario Sideri, Francesca Maria Carozzi, Amina Vocaturo, Franco Maria Buonaguro, Maria Lina Tornesello, Elena Burroni, Luciano Mariani, Sara Boveri, Leandra Maria Zaffina, Francesco Chini

**Affiliations:** 1 AUSL Reggio Emilia, via Amendola 2, Reggio Emilia, 42122, Italy; 2 European Institute of Oncology, Milan, Italy; 3 ISPO Toscana, Florence, Italy; 4 National Cancer Institute Regina Elena, Rome, Italy; 5 National Cancer Institute Fond Pascale, Naples, Italy; 6 Agency for Public Health, Lazio Region, Rome, Italy

## Abstract

**Objective:**

The aim of this study is to describe the prevalence of HPV types in invasive cervical cancers in Italy from 1996 to 2008.

**Methods:**

A pooled analysis of the three largest case series typed to date was performed. HPV typing was performed on paraffin-embedded slices. Molecular analyses were performed in four laboratories. Multivariate analyses were performed to test the associations between calendar time, age, and geographical area and the proportion of types 16/18.

**Results:**

Out of 574 cancers, 24 (4.2%) were HPV negative. HPV 16 and 18 were responsible for 74.4% (378/508) and 80.3% (49/61) of the squamous cancers and adenocarcinomas, respectively. Other frequent types were 31 (9.5%), 45 (6.4%), and 58 (3.3%) for squamous cancers and 45 (13.3%), 31, 35, and 58 (5.0%) for adenocarcinomas. The proportion of HPV 16 and/or 18 decreased with age (p-value for trend <0.03), while it increased in cancers diagnosed in more recent years (p-value for trend < 0.005).

**Conclusions:**

The impact of HPV 16/18 vaccine on cervical cancer will be greater for early onset cancers. In vaccinated women, screening could be started at an older age without reducing protection.

## Background

Cervical cancer is still the second leading cancer in women worldwide, with 585,278 new cases estimated in 2010. More than 80% of these cases occurred in low-medium income countries [1,2]. In Italy, the incidence has been decreasing over the last few decades and now only 2800 new cases per year (8.2/100000 EUR STD) are estimated [3].

It has been demonstrated that HPV infection is a necessary cause for cervical cancer [4]. In particular, 12 high-risk types [5] cause about 90% of all cases.

The prevalence of HPV types infecting the lower genital tract in the healthy population shows geographical variations [6]. Instead, the prevalence of HPV types in cancer is much more stable across geographic areas: type 16 is always the most represented, followed by 18, 45, 31, and 33 [7,8].

Nevertheless, some differences among continents have been consistently observed: In Africa and Eastern Asia, the proportion of cancers due to HPV types 16 and 18 is slightly lower than in Western Europe and North America [7,8]. Furthermore, early onset cancers are more frequently linked to HPV 16, 18, and 45 [7,9].

Many hypotheses have been formulated to explain these differences. The one most commonly accepted to explain the higher proportion of type 16 in early onset cancers is that this type has a greater potential to transform the cell compared to other high-risk HPV types and consequently there is a higher probability of progression from infection to cancer in a relatively short time. There is evidence from prospective cohort studies of shorter time to progression from HPV16 infection to high-grade Cervical Intraepithelia Neoplasia (CIN 3) [10-12]. However, this cannot be demonstrated for cervical cancer since CIN 3 and even CIN 2 must be treated once detected.

Data from cohort studies on time to progression for types 18 and 45 are less consistent [10,11] because these types are quite uncommon in the general population and their higher cancerogenicity has been deduced by in vitro transformation data as well as by epidemiological case–control and cross-sectional studies.

The prevalence of cytological screening uptake in the population strongly influences the epidemiology of cervical cancer. In highly screened populations, Pap test’s greater efficacy in detecting squamous precancerous lesions than glandular lesions leads to marked reduction in the incidence in the former [13,14] and smaller reduction in the latter [15-17]. Furthermore, cytological screening shows higher efficacy in decreasing the incidence in women 30 years and older [18]. Consequently, the ratio between early onset and late onset and between squamous cancers and adenocarcinomas changes in screened population.

## Objective

The aim of this work is to describe the prevalence of HPV types in Italian invasive cervical cancers from 1996 to 2008. The study is based on the pooling of three cases large series [9,19,20].

In particular, we describe the associations between ca lendar time, age, and geographical area and the proportion of vaccine-targeted types (16 and 18) and early onset types (types 16/18/45).

## Methods

### Study population, selection cases

Histologically confirmed cervical cancer diagnoses from three large series were included in the study. All three studies performed *ad hoc* morphological and molecular analyses on archival paraffin-embedded invasive cervical cancer (ICC) specimens.

Central and Southern Italy Study [9]: 193 cases from eight centers (S. Giovanni Hospital in Rome and Belcolle Hospital in Viterbo, in Lazio; National Cancer Institute Fondazione Pascale in Naples, Campania; Atri, Abruzzo; Catania, Sicily; Cagliari, Sardinia; ISPO and S. Maria Annunziata Hospital in Florence, Tuscany) diagnosed between 1999 and 2008.

Rome Study [19]: 134 cases between 2001 and 2006, mostly in residents in central Italy, identified at and retrieved from the Regina Elena Cancer Institute in Rome.

Milan Study [20]: 268 cases from all over the country - Milan and surrounding area (n = 57, 21.3%), northern Italy (n = 81, 30.2%), central Italy (n = 64, 23.9%), and southern Italy (n = 66, 24.6%) - referred to the European Institute of Oncology between 1996 and 2006.

### Pathology and laboratory procedure

Paraffin block selection and microtome sectioning, performed under strict conditions to avoid potential contamination, employed a sandwich technique to obtain 5 or 10-μm tissue section from each paraffin block. First and last microtome sections were stained for histopathological evaluation, while the internal sections were processed for DNA extraction. In one centre (Naples), morphologic check showed that the mean percentage of tumor cell staining was 64% (range: 5-100%), with a relevant proportion of sample slices containing a limited quantity of neoplastic tissue within a healthy tissue context.

No central histologic review was planned for this study.

### DNA extraction and HPV typing

Molecular analyses were performed in four centers: the Milan Study sent specimens to the International Agency for Research on Cancer (IARC, Lyon, France) [20], the Rome Study to the Catalan Institute of Oncology-ICO Survey [19], and the Central and Southern Italy Study to the laboratory at ISPO in Florence and to the Pascale National Cancer Institute in Naples [9].

For each sample, DNA concentration was evaluated by spectrophotometry and expressed in ng/μL.

HPV genotyping was done using different PCR-based strategies in a well-defined algorithm.

#### The Central and Southern Italy study

A PCR assay based on GP5+/GP6+ primers was used (Consensus High Risk HPV Genotyping kit, Digene, Qiagen). All samples and controls were subjected to the reverse line blot (RLB) for detection of 12 high-risk HPV types (16, 18, 31, 33, 35, 39, 45, 51, 52, 56, 58, and 59), 1 probably carcinogenic HPV type (68), and 5 possibly carcinogenic HPV types (26, 53, 66, 73, and 82) [9]. To amplify negative samples and to overcome any false negatives due to inhibitors commonly present in formalin-fixed samples or due to low copy number of HPV DNA, a sequence of steps was carried out [9].

#### The Rome study

For genotyping HPV DNA, 10 μl of the PCR amplimers of the HPV DNA-positive samples, as identified by DEIA, were analyzed by Line Probe Assay-LiPA25 version 1 (Labo Biomedical Products, Rijswijk ,The Netherlands), which can detect 25 different high-risk and low-risk HPV types (6, 11, 16, 18, 31, 33, 34, 35, 39,40, 42, 43, 44, 45, 51, 52, 53, 54, 56, 58, 59, 66, 68, 70,74) [19].

#### The Milan study

Multiplex PCR was performed using the Qiagen Multiplex PCR Kit according to the manufacturer’s instructions, with a mixture containing 19 pairs of HPV-type specific primers (type 16, 18, 26, 31, 33, 39, 45, 51, 52, 53, 56, 58, 59, 66, 68, 70, 73, 82) following the procedure described by Gheit et al. [21]. To increase the sensitivity of HPV16 detection, the PCR forward primer for HPV16 E7 was modified as described by Cazzaniga et al. [22].

### Statistical analysis

Our tables show the type distribution found in squamous and cervical cancers. We also present the proportion of types 16 and 18 or 16, 18, and 45 by age, calendar time at diagnosis, and geographic area. Proportions with relative 95% confidence intervals were computed. Multivariate models were constructed to identify determinants of the proportion of vaccine-covered types (16 and 18) and early onset types (16, 18 and 45). All the analyses were performed using STATA 11.0.

## Results

After histological evaluation, 595 ICC specimens from the original studies were included. Of these, 4 cases from the Rome Study and 17 from the Milan Study were excluded from the present study because the information about typing was missing. Thus, 574 cases were available for the analyses.

The age range of the women included in this study was 23–85 years (mean age 52.4 years).

Squamous cell carcinoma was the most represented histotype (508 cases: 88.5%), followed by adenocarcinoma (55 cases: 9.6%), then adenosquamous cell carcinoma (6 cases: 1.0%), and other minor histotypes (5 cases: 0.9%). For all the following analyses, adenosquamous cell carcinomas were grouped with adenocarcinomas.

Adenocarcinoma was not associated with age at diagnosis (p = 0.57), but a strong time trend, with increasing proportion of adenocarcinomas in the recent years, was observed (p-value for trend <0.002).

Twenty-four (4.2%) cases were HPV DNA-negative. Among the HPV-positive cases, 74 (13.4%) were multiple infections, and 7 cases were non-typed.

HPV 16 was the most frequent type detected, alone or in coinfection with other types: 69.3% and 58.3% in squamous and adenocarcinomas, respectively (Table 1).

**Table 1:** HPV types by histotype

	**Squamous cell carcinoma**		**Adenocarcinoma and Adenosquamous cell carcinoma**		Total*	
ALL	508		61		574	
	N	%	N	%	N	%
HPV negative	23	4.5	1	1.6	24	4.2
HPV types						
*6*	2	0.4	0	0.0	2	0.4
*16*	336	69.3	35	58.3	373	67.8
*18*	42	8.7	14	23.3	59	10.7
*31*	46	9.5	3	5.0	49	8.9
*33*	14	2.9	0	0.0	14	2.5
*35*	9	1.9	3	5.0	12	2.2
*39*	5	1.0	0	0.0	5	0.9
*42*	2	0.4	0	0.0	2	0.4
*45*	31	6.4	8	13.3	39	7.1
*51*	7	1.4	0	0.0	7	1.3
*52*	9	1.9	0	0.0	9	1.6
*53*	3	0.6	1	1.7	4	0.7
*56*	5	1.0	1	1.7	6	1.1
*58*	16	3.3	3	5.0	19	3.5
*59*	5	1.0	1	1.7	6	1.1
*66*	5	1.0	0	0.0	5	0.9
*67*	1	0.2	0	0.0	1	0.2
*68*	3	0.6	0	0.0	3	0.5
*70*	4	0.8	0	0.0	4	0.7
*73*	9	1.9	0	0.0	9	1.6
*82*	1	0.2	0	0.0	1	0.2
*not typed*	4	0.8	3	5.0	7	1.3

*including five other minor histotypes; three cases with HVP type 18 and two cases with HVP type 16.

HPV 18 was the second most frequent in adenocarcinomas (23.3%), and third in squamous (8.7%; p-value = 0.0003). The cumulative proportion of HPV 16 and/or HPV 18 was slightly higher in adenocarcinomas than in squamous cell carcinomas, though not significantly (81.8% vs 74.4%, p-value = 0.2). HPV 45 was also more frequent in adenocarcinomas than in squamous (13.3% vs 6.4%, p-value = 0.04), while HPV 31 was more frequent in squamous cell carcinomas, though not significantly so (9.5% vs 5.0%). The fifth most frequent type was HPV 58 (3.3% and 5.0% in squamous and adenocarcinomas, respectively). HPV 33 ranked sixth (2.9% in squamous and null in adenocarcinomas), and HPV 35 ranked seventh (1.9% and 5% in squamous and adenocarcinomas, respectively).

There was no significant difference in the geographical distribution of HPV 16 and 18, whereas we observed a significant (p-value = 0.03), higher proportion of HPV 31 in northern Italy (13.1%) than in central and southern Italy (7.5% and 6.8%, respectively) (Table 2).

**Table 2:** HPV types by Italian geographical area

	**north**		**centre**		**south and islands**		**abroad & missing**		**Total**	
ALL	154		193		205		22		574	
	N	%	N	%	N	%	N	%	N	%
HPV negative	1	0.6	6	3.1	14	6.8	3	13.6	24	4.2
HPV types										
*6*	0	0.0	1	0.5	1	0.5	0	0.0	2	0.4
*16*	100	65.4	127	67.9	134	70.2	12	63.2	373	67.8
*18*	19	12.4	18	9.6	22	11.5	1	5.3	60	10.9
*31*	20	13.1	14	7.5	13	6.8	2	10.5	49	8.9
*33*	6	3.9	3	1.6	4	2.1	1	5.3	14	2.5
*35*	4	2.6	2	1.1	5	2.6	1	5.3	12	2.2
*39*	2	1.3	2	1.1	1	0.5	0	0.0	5	0.9
*42*	0	0.0	1	0.5	1	0.5	0	0.0	2	0.4
*45*	9	5.9	16	8.6	12	6.3	2	10.5	39	7.1
*51*	3	2.0	1	0.5	2	1.0	1	5.3	7	1.3
*52*	5	3.3	3	1.6	1	0.5	0	0.0	9	1.6
*53*	2	1.3	2	1.1	0	0.0	0	0.0	4	0.7
*56*	2	1.3	3	1.6	1	0.5	0	0.0	6	1.1
*58*	5	3.3	8	4.3	6	3.1	0	0.0	19	3.5
*59*	3	2.0	0	0.0	3	1.6	0	0.0	6	1.1
*66*	1	0.7	2	1.1	2	1.0	0	0.0	5	0.9
*67*	0	0.0	1	0.5	0	0.0	0	0.0	1	0.2
*68*	1	0.7	0	0.0	2	1.0	0	0.0	3	0.5
*70*	2	1.3	0	0.0	1	0.5	1	5.3	4	0.7
*73*	5	3.3	2	1.1	2	1.0	0	0.0	9	1.6
*82*	1	0.7	0	0.0	0	0.0	0	0.0	1	0.2
*not typed*	0	0.0	3	1.6	4	2.1	0	0.0	7	1.3

There was a significant decreasing trend of HPV 16 and 18 in proportion with age, dropping from 94% to about 75% after the age of 35 (p-value = 0.03). The trend was even stronger when we included HPV 45 (p-value = 0.005) (Table 3).

**Table 3:** HPV types 16/18 and HPV types 16/18/45 by histotype, study and age

		***<=24***	***25-34***	***35-44***	***45-54***	***55-64***	***65+***	***All ages***
**Histological type**								
**Squamous cell carcinoma**	**16/18 (n)**	1	40	108	85	61	69	364
	*%*	*100,0*	*93,0*	*76,1*	*72,6*	*72,6*	*75,0*	*76,0*
	**16/18/45 (n)**	1	41	118	94	64	72	390
	*%*	*100*	*95,3*	*83,1*	*80,3*	*76,2*	*78,3*	*81,4*
	**Any type (n)**	1	43	142	117	84	92	479
**Adeno- and Adenosquamous cell carcinoma**	**16/18 (n)**	0	5	15	5	10	8	43
	*%*	*0*	*100*	*93,8*	*55,6*	*83,3*	*61,5*	*76,8*
	**16/18/45 (n)**	1	5	15	6	11	11	49
	*%*	*100*	*100*	*93,8*	*66,7*	*91,7*	*84,6*	*87,5*
	**Any type (n)**	1	5	16	9	12	13	56
**Study**								
**Milan Study**	**16/18 (n)**	0	25	65	44	38	15	187
	*%*	*-*	*89,3*	*75,6*	*71,0*	*73,1*	*71,4*	*75,1*
	**16/18/45 (n)**	0	26	70	49	38	16	199
	*%*	*-*	*92,9*	*81,4*	*79,0*	*73,1*	*76,2*	*79,9*
	**Any type (n)**	0	28	86	62	52	21	249
**Rome Study**	**16/18 (n)**	1	9	25	14	16	15	80
	*%*	*100,0*	*100,0*	*69,4*	*60,9*	*84,2*	*62,5*	*71,4*
	**16/18/45 (n)**	1	9	28	17	16	15	86
	*%*	*100,0*	*100,0*	*77,8*	*73,9*	*84,2*	*62,5*	*76,8*
	**Any type (n)**	1	9	36	23	19	24	112
**Central and Southern Italy Study**	**16/18 (n)**	0	11	33	32	17	47	140
	*%*	*0,0*	*100,0*	*91,7*	*78,0*	*68,0*	*78,3*	*80,5*
	**16/18/45 (n)**	1	11	35	34	21	52	154
	*%*	*100,0*	*100,0*	*97,2*	*82,9*	*84,0*	*86,7*	*88,5*
	**Any type (n)**	1	11	36	41	25	60	174
**Total**	**16/18 (n)**	2	46	124	91	71	78	412
	*%*	*66,7*	*93,9*	*78,0*	*71,7*	*74,0*	*73,6*	*76,3*
	**16/18/45 (n)**	3	47	134	101	75	84	444
	*%*	*100*	*95,9*	*84,3*	*79,5*	*78,1*	*79,2*	*82,2*
	**Any type (n)**	3	49	159	127	96	106	540

*including five other minor histotypes; three cases with HPV type 18 and two cases with HPV type 16; one case in each age class except 55–64 years old.

There was also a significant trend in the proportion of types 16 and 18 with calendar time, with a higher proportion in the more recent years (p-value = 0.005) (Table 4), with no heterogeneity among centres.

**Table 4:** HPV types 16/18 and HPV types 16/18/45 by histotype, study and year of diagnosis

		***1999°***	***2000***	***2001***	***2002***	***2003***	***2004***	***2005***	***2006***	***2007***	***2008***	***All years***
**Histological type**												
**Squamous cell carcinoma**	**16/18 (n)**	16	23	30	34	56	60	62	62	13	9	365
	*%*	*64,0*	*74,2*	*73,2*	*66,7*	*73,7*	*76,9*	*75,6*	*89,9*	*81,3*	*81,8*	*75,9*
	**16/18/45 (n)**	18	24	30	37	64	65	68	63	14	9	392
	*%*	*72,0*	*77,4*	*73,2*	*72,5*	*84,2*	*83,3*	*82,9*	*91,3*	*87,5*	*81,8*	*81,5*
	**Any type (n)**	26	31	41	51	76	78	82	69	16	11	481
**Adeno- and Adeno-squamous cell carcinoma**	**16/18 (n)**	0	2	1	3	6	5	9	10	7	0	43
	*%*	-	*50,0*	*50,0*	*75,0*	*85,7*	*100,0*	*75*	*83,3*	*70*	-	76,8
	**16/18/45 (n)**	0	2	2	4	6	5	11	11	8	0	49
	*%*	*-*	*50,0*	*100,0*	*100,0*	*85,7*	*100,0*	*91,7*	*91,7*	*80*	*-*	*87,5*
	**Any type (n)**	0	4	2	4	7	5	12	12	10	0	56
**Study**												
**Milan Study**	**16/18 (n)**	11	20	19	22	28	24	33	29	1	0	187
	*%*	*61,1*	*71,4*	*70,4*	*71,0*	*68,3*	*85,7*	*82,5*	*85,3*	*100,0*	*-*	*75,1*
	**16/18/45 (n)**	12	21	19	23	32	24	36	31	1	0	199
	*%*	*66,7*	*75,0*	*70,4*	*74,2*	*78,0*	*85,7*	*90,0*	*91,2*	*100,0*	*-*	*79,9*
	**Any type (n)**	19	28	27	31	41	28	40	34	1	0	249
**Rome Study**	**16/18 (n)**	0	0	7	8	16	22	16	11	0	0	80
	*%*	*-*	*-*	*100,0*	*50,0*	*76,2*	*71,0*	*66,7*	*84,6*	*-*	*-*	*71,4*
	**16/18/45 (n)**	0	0	7	10	17	24	17	11	0	0	86
	*%*	*-*	*-*	*100,0*	*62,5*	*81,0*	*77,4*	*70,8*	*84,6*	*-*	*-*	*76,8*
	**Any type (n)**	0	0	7	16	21	31	24	13	0	0	112
**Central and Southern Italy Study**	**16/18 (n)**	5	5	5	7	18	19	22	32	19	9	134
	*%*	*71,4*	*71,4*	*55,6*	*87,5*	*85,7*	*79,2*	*73,3*	*94,1*	*76,0*	*81,8*	*76,1*
	**16/18/45 (n)**	6	5	6	8	21	22	26	32	21	9	156
	*%*	*85,7*	*71,4*	*66,7*	*100,0*	*100,0*	*91,7*	*86,7*	*94,1*	*84,0*	*81,8*	*88,6*
	**Any type (n)**	7	7	9	8	21	24	30	34	25	11	176
**Total Squamous cell carcinoma**	**16/18 (n)**	16	25	31	37	65	66	72	72	20	9	413
	*%*	*64,0*	*74,2*	*73,2*	*66,7*	*73,7*	*76,9*	*75,6*	*89,9*	*81,3*	*81,8*	*75,9*
	**16/18/45 (n)**	18	26	32	41	73	71	80	74	22	9	446
	*%*	*72,0*	*77,4*	*73,2*	*72,5*	*84,2*	*83,3*	*82,9*	*91,3*	*87,5*	*81,8*	*81,5*
	**Any type (n)**	26	35	43	55	86	84	95	81	26	11	542

*including five other minor histotypes; three cases with HPV type 18 and two cases with HPV type 16; Three cases diagnosed in 2003, one in 2004 and one in 2005.

°Including one case occurred in 1998.

The multivariate model shows that the effects of age and calendar time are independent and that they are not due to any bias in center recruitment and laboratory techniques adopted for typing. The same results are obtained if we limit the analyses to squamous cell cancers (Table 5); stratified analyses for adenocarcinoma were not performed due to small numbers.

**Table 5:** Multivariate models

**Any Histotype**				
**HPV types 16/18**	OR*	**95% CI**		**P-value**
age	0.98	0.97	1.00	0.030
year	1.16	1.05	1.29	0.005
*§including histotype in the covariates has no effect (p-value = 0.9)*				
**HPV types 16/18/45**	OR*	**95% CI**		**P-value**
age	0.98	0.96	0.99	0.005
year	1.17	1.04	1.31	0.008
*§including histotype in the covariates has no effect (p-value = 0.7)*				
**Squamous cell carcinoma**				
**HPV types 16/18**	OR*	**95% CI**		**P-value**
age	0.99	0.97	1.00	0.076
year	1.16	1.04	1.30	0.008
**HPV types 16/18/45**	OR*	**95% CI**		**P-value**
age	0.98	0.96	0.99	0.006
year	1.17	1.03	1.32	0.012
*Adjusted by study				

## Discussion

These three large Italian case series confirm some previously published results [7,8]. The proportion of HPV-positive cases (96.0%) is slightly higher compared with that recently published by IARC (92.5%), meaning that the average quality of specimens and laboratory procedures was good.

Again, in line with de Sanjose’s study, HPV 16 and 18 were detected in about 77% of cervical cancers [7]. This proportion is higher than that observed in preinvasive lesions (CIN2 and CIN3) in the same populations [9,23], which is consistent with findings in almost all the previous studies [24,25]. The proportions of HPV 18 and 45 are higher in adenocarcinomas than in squamous cancers, as previously reported in larger studies [7,8,26].

It is interesting that HPV 33 did not rank in the top five, in contrast with the worldwide distribution. Other studies have already reported higher prevalence of HPV 31 than 33 in southern Europe [8].

In our series, more than 1 out of 9 cases were adenocarcinomas, which is typical of high-moderate screening coverage [27], but the proportion of adenocarcinomas showed an increasing time trend during the study period, as expected, since Pap test coverage increased over the same period [28].

The main objective of this paper was to describe changes in the proportion of vaccine-covered HPV types 16 and 18 and in early onset HPV types 16, 18, and 45, according to the geographical area, age, and the calendar time.

Only few differences among geographic areas emerged and, even when statistically significant, they may have been due to chance, given the enormous number of statistical tests that can be performed comparing these distributions.

The most remarkable results in our analyses were the trends in HPV 16, 18, and 45 with age and calendar time. The multivariate analysis clearly shows that the two trends are independent and not due to reciprocal confounding, to the increase in adenocarcinomas proportion in recent years, or to different PCR techniques adopted for typing in the three studies.

The relation with age was expected since these are known to be early onset types [7,9,20,29], particularly HPV16, which showed a higher probability of progression [10,11]. In fact, if we assume a multi-step model in cancer progression, a higher probability of progression will produce a higher proportion of cases in younger ages, as sexual activity (and with it, possible infection) has only recently begun. Differently from the large study by de Sanjose and collaborators [7], our trend was due to an excess of HPV 16, 18 and 45 HPV-related squamous cancer in women below age 35, but we did not have enough statistical power to accurately describe the shape of the curve.

On the contrary, the association with calendar time was not observed in other large series [30,31] and we must therefore assume it is a context-specific finding. It must be noted that the screening coverage in Italy has increased over the last decade, from 50% in 1994 to 73% in 2004. The increase in screening coverage has the immediate effect of decreasing the overall incidence of cervical cancer, as illustrated by the dramatic decrease in incidence in Italy, from 9.2/100.000 in 1996 to 7.7/100.000 in 2005 [28]. However, Pap-test preventive efficacy is not the same for all the cancers. Indeed, it is well known that the efficacy of screening is stronger for squamous cancers than for adenocarcinomas [15-17], and it has recently been demonstrated that the efficacy is very low for early onset cancers [18]. In line with these observations, screening might be less effective for those cancers caused by HPV 16, 18 and 45, virus types known to be associated with early onset. Consequently, in a highly screened population, the few cases occurring are in part those occurring in the under-screened or not covered minority. Further, a relevant proportion is also made up of the few cases occurring during the interval between two screening episodes. The cases escaping the Pap-test control, according to this hypothesis, are more likely to be linked to HPV 16, 18, or 45.

The possibility of a more rapid progression associated with HPV 16 and its variants is supported by the Guanacaste Cohort Study, a 7-year nested case–control study conducted on 10,049 women [32]. The study showed that HPV 16 viruses, particularly non-European HPV 16 variants (mainly Asian American, AA), are significantly associated with higher risk of developing invasive cervical cancer [OR, 6.3 (95% CI, 1.6–24.6)]. Similar data have also been reported for HPV 16 AA variants in the Italian population, with a relative risk of 24.5 of developing invasive cervical cancer [33].

Another hypothesis is that HPV 16/18/45-related cancer precursors occur at a younger age, are far superior in number in comparison with other high-risk HPV-related cancer precursors, are not easily detected by cytology screening, and the transit time from precursor to cancer is not necessarily quicker. Recent population-based studies have shown that by using very sensitive screening tests such as HPV DNA testing, the number of precancerous lesions missed by cytology proves to be considerable [34]. This lack of cytology screening sensitivity is particularly evident in the younger age groups, where HPV testing may detect high-grade CINs more frequently than cytology [34]. It is already known that high-grade CINs occurring at a young age are mostly related to HPV 16/18 [9,29]; the trend in distribution of HPV genotypes in invasive cancers in different calendar years observed in the present study may therefore be the result of the faster and more frequent formation of high-grade CINs in young girls and the inability of cytology screening to detect most of these lesions, typically related to HPV 16/18/45. The large number of high-grade CINs left in situ could then generate invasive lesions between two screening intervals more frequently than would the other high-risk genotypes, whose ability to persist and generate CIN3 lesions is less efficient than HPV16 and, albeit less markedly, than 18.

Both these hypotheses consider the three HPV types related to early onset cancers as a unique group, but the evidence available about speed of progression from infection to CIN3 and cancer and about screening sensitivity are different for HPV 16 and HPV 18, and are almost absent for HPV 45.

In conclusion, the proportion of HPV 16 and 18 types may be higher in highly screened populations because these types more frequently escape Pap test control. Consequently, the dramatic decrease in cancer incidence after screening introduction may be slightly less relevant for HPV 16- and 18-related cancers. Unfortunately, the effectiveness of Pap test as prevention cannot easily be increased by shortening screening intervals; a large body of evidence shows that reducing the interval from 3 years to 1 has a small effect on cancer incidence and a strong effect on the number of screening-induced treatments [35,36]. On the contrary, by shifting to HPV as primary screening test, we can imagine a protocol that increases protection and decreases over-treatment, adopts longer intervals for HPV-negative women, and modulates the follow up for HPV-positive women according to the genotype.

### Limits

As the present study has several limits, we can only confirm previously observed data or formulate hypotheses to be confirmed in other studies.

The first limitation is that we did not perform a central pathology review, even if all the cases were specifically reviewed for the original study in each center [9,19,20].

As typing was performed on archival paraffin-embedded samples, some trends could be due to reduced analytical accuracy of typing methods on older samples, but this is not very likely to change the proportion of types.

Our archives do not report some of the information that might have been crucial to testing our hypothesis. In particular, we do not know if the cases had had a previous negative Pap test. Furthermore, we do not know the stage at diagnosis. It could be extremely interesting to identify the micro-invasive cancers (FIGO IA1), since these cancers are virtually all asymptomatic and are thus mostly screen-detected [37].

Finally, our series do not come from population-based registries and thus may not represent the universe of cancers in Italy. Furthermore, in some of the areas with higher screening coverage, i.e., northern Italy and Rome, the participating centers are not reference centre for screening programs, while in the areas with lower screening coverage, i.e. southern Italy, they are. It is likely that the proportion of screening-detected cancers in our archives does not represent the proportion in the cancer population in each geographic area. This may explain why we did not observe an effect of the geographic area on the proportion of HPV 16, 18, or 45, despite the broad differences in screening coverage among the areas.

## Conclusions

The impact of HPV 16/18 vaccine on cervical cancer will be proportionally more relevant for early onset cancers. Considering the very low overall risk in women under age 35 and that the proportion of vaccine-targeted cancers is over 90% in that age group, it may be necessary to postpone starting age for screening in vaccinated cohorts in order to reduce the risk of over-treatment and to contain costs.

Furthermore, we suggest that screening is less effective in preventing the incidence of HPV 16 and 18 invasive cancers, given their shorter sojourn time in the precancerous stages. If this is true, then the screening interval in vaccinated women should take into account the longer sojourn time of CIN2 and CIN3 lesions due to non-16/18 HPV types.

### Ethical Approval

This is a pooled analysis of already published studies. All the three studies were approved by Ethic review board initally. This pooled analysis has been financed by the Ministry of Health and it was planned in the protocol of the most recent of the three studies [9].

## Appendix

### HPV prevalence Italian working group

Abruzzo: Angeloni Claudio, Lattanzi Amedeo, Maccallini Vincenzo, Caraceni Donatella, Fortunato C.

Cagliari: Macis Rosalba, Pilia Massimo, Caredda Valeria.

Campania: Carillo Giuseppe, Di Iasi Angela, Santarsiere Aldo, Casto Loredana, Manno Maria, Santangelo Claudia, Pini Maria Teresa, Gallicchio Giuseppina, Scherillo Isabella, Barretta Elena, De Santis Vincenzo, Ercole Filomena.

Catania: Scalisi Aurora, Spampinato Pina, Cantarella Maria Antonietta, Miano Maria Giuseppina.

Lazio: Giorgi Rossi Paolo, Chini Francesco, Guasticchi Gabriella, Capparucci Paola, Marsili Laila Maria, Tufi Maria Concetta, Gomez Vito, Verrico Giovanna, Schiboni Maria Luisa, Pellegrini Antonella, Bove Emilia, D’Addetta Albina, Placidi Antonio.

National Cancer Institute Fond Pascale, Naples: Buonaguro Franco Maria, Tornesello Maria Lina, Loquercio Giovanna, Losito Simona, Botti Gerardo, Vecchione Aldo.

European Institute of Oncology, Milan: Sideri Mario, Boveri Sara

Tuscany: Carozzi Francesca M., Confortini Massimo, Bisanzi Simonetta, Sani Cristina, Venturini Giulia, Burroni Elena, Tinacci Galliano.

National Cancer Institute Regina Elena, Rome: Mariani Luciano, Vocaturo Amina, Benevolo Maria, Marandino Fernando.

Ministry of Health: Antonio Federici.

## Competing interests

F.M. Carozzi is an occasional advisor to Gen-Probe, Abbott, Sanofi, and GlaxoSmithKline.

## Authors’ contributions

PGR, MS, FMC, MLT, FMB and LM conceived the study and planned the analyses. PGR, FMC, FMB, MS, and AV coordinated the sample collection. MLT, MLT, AV, and EB coordinated and performed the laboratory tests. FC planned the data collection and performed the statistical analyses. PGR drafted the paper.0020. All authors read and approved the final manuscript.
